# Updated findings on temporal variation in radiation-effects on cancer mortality in an international cohort of nuclear workers (INWORKS)

**DOI:** 10.1007/s10654-024-01178-6

**Published:** 2024-11-22

**Authors:** Robert D. Daniels, Stephen J. Bertke, Kaitlin Kelly-Reif, David B. Richardson, Richard Haylock, Dominique Laurier, Klervi Leuraud, Monika Moissonnier, Isabelle Thierry-Chef, Ausrele Kesminiene, Mary K. Schubauer-Berigan

**Affiliations:** 1 National Institute for Occupational Safety and Health (NIOSH), 1090 Tusculum Avenue, Mailstop 12, Cincinnati, OH 45226 USA; 2 Department of Environmental and Occupational Health, Program in Public Health, University of California, Irvine, CA USA; 3 UK Health Security Agency, Chilton, Didcot, Oxfordshire UK; 4 Institute for Radiological Protection and Nuclear Safety (IRSN), Fontenay-aux-Roses, France; 5 International Agency for Research on Cancer (IARC/WHO), Lyon, France; 6 Barcelona Institute for Global Health (ISGlobal), Barcelona, Spain

**Keywords:** Cancer, Epidemiology, Longitudinal studies, Dose-response, Mortality studies

## Abstract

The International Nuclear Workers Study (INWORKS) contributes knowledge on the dose-response association between predominantly low dose, low dose rate occupational exposures to penetrating forms of ionizing radiation and cause-specific mortality. By extending follow-up of 309,932 radiation workers from France (1968–2014), the United Kingdom (1955–2012), and the United States (1944–2016) we increased support for analyses of temporal variation in radiation-cancer mortality associations. Here, we examine whether age at exposure, time since exposure, or attained age separately modify associations between radiation and mortality from all solid cancers, solid cancers excluding lung cancer, lung cancer, and lymphohematopoietic cancers. Multivariable Poisson regression was used to fit general relative rate models that describe modification of the linear excess relative rate per unit organ absorbed dose. Given indication of greater risk per unit dose for solid cancer mortality among workers hired in more recent calendar years, sensitivity analyses considering the impact of year of hire on results were performed. Findings were reasonably compatible with those from previous pooled and country-specific analyses within INWORKS showing temporal patterns of effect measure modification that varied among cancers, with evidence of persistent radiation-associated excess cancer risk decades after exposure, although statistically significant temporal modification of the radiation effect was not observed. Analyses stratified by hire period (< 1958, 1958+) showed temporal patterns that varied; however, these analyses did not suggest that this was due to differences in distribution of these effect measure modifiers by hire year.

**Supplementary Information:**

The online version contains supplementary material available at 10.1007/s10654-024-01178-6.

## Introduction

The International Nuclear Workers’ Study (INWORKS) is a longstanding collaboration coordinated by the International Agency for Research on Cancer (IARC) to examine mortality in a cohort of radiation workers employed in France, the United Kingdom (UK), and the United States (US) [1–12]. These workers have been studied in earlier pooled analyses [13–19] and country-specific analyses, e.g [20–24]. Recent analyses reported positive associations between: colon absorbed dose (in gray, Gy) and all solid cancers [excess relative rate (ERR) Gy^–1^ = 0.52; 90% confidence interval (CI): 0.27, 0.77] and all solid cancers excluding lung (ERR Gy^–1^ = 0.46; 90% CI 0.18, 0.76); lung dose and lung cancer (ERR Gy^–1^ = 0.67; 90% CI 0.21, 1.19); and red bone marrow (RBM) dose and leukemia excluding chronic lymphocytic leukemia (CLL) (ERR Gy^–1^ = 2.68; 90% CI 1.13, 4.55), among others [1, 11, 12].

Previous INWORKS analysis found evidence suggesting variation in ERR Gy^-1^ by temporal factors that differed by cancer type, although significant effect modification was observed only for chronic myeloid leukemia (CML), which varied by time since exposure (TSE) [4]. That study also found persistent and late-onset radiation risk for certain cancers, suggesting that additional follow-up was needed to fully describe lifetime risk. Therefore, the current study examines effect modification by TSE, age at exposure (AE), and attained age (AA) after extending follow-up, adding about 2.5 million person-years and over 10,000 additional solid cancer deaths.

## Methods

### Study cohort

The cohort is described elsewhere [8]. Briefly, it comprises 309,932 nuclear workers employed for one or more years in radiation work and monitored for exposure to ionizing radiation. Information was obtained from French employers (Commissariat à l’Energie Atomique et aux énergies alternatives, Orano, and Electricité de France), the UK National Registry for Radiation Workers (NRRW, including information provided by the Atomic Weapons Establishment, British Nuclear Fuels, UK Atomic Energy Authority, British Energy Generation, Magnox Electric, and the Ministry of Defence, among others), and from the US Department of Energy’s Hanford site, Savannah River site, Oak Ridge National Laboratory (ORNL), and Idaho National Laboratory, as well as from the Portsmouth Naval Shipyard.

Vital status was ascertained via linkage with national and regional death registries, employment records, tax records, and social security administration records. Follow-up was through 2012, 2014, and 2016 for the UK, French, and US cohorts, respectively [1, 11, 12]. Observation began on the date first monitored, the start date of the applicable death registry, or one year after date first hired, whichever was latest. The observation period ended on the earliest of the death date, date lost to follow-up, or the end of follow-up. Cancer deaths were determined from the underlying cause of death from death certificates typically coded to the revision of the International Classification of Diseases (ICD) in effect at the time of death (Online Resource Table S1).

## Outcomes of interest

Analyses were first restricted to previously examined sites with either 150 or more deaths or evidence of significant temporal effect modification [4]. This list included all solid cancers (ICD 10 C00-C80, C97 except C46.3); cancers of the lung, trachea, and bronchus (ICD 10 C33–C34); non-CLL leukemia (ICD 10 C91.0, C91.2–C91.7, C92.0–C95.0, and C95.2–C95.7); acute myeloid leukemia (AML; ICD 10 C92.0, C92.3–C92.6, C93.0, C94.0, C94.2, C94.4, C94.5), CML (ICD 10 C92.1, C93.1, and C94.8); non-Hodgkin lymphoma (NHL; ICD 10 C82–C85, C88, and C96); and multiple myeloma (MM; ICD 10 C90). Myelodysplastic syndrome (MDS, ICD 10 D46) in combination with AML was included given its potential to progress to AML. Lastly, all solid cancers excluding lung cancer was included following comments received during review (Online Resource Table S1). As in previous analyses, CLL was excluded given an absence of evidence on radiogenicity [4].

## Exposure

Details on dose reconstruction are presented elsewhere [9, 18]. Briefly, recruitment of workers focused on nuclear facilities where most dose stemmed from external sources of penetrating photon radiation, and measurements from personal monitoring were reasonably available throughout the study period. Extensive efforts were made to abstract information from dosimetry records on all workers by dosimetrists blinded to case status. Recorded doses from personal monitoring were adjusted to account for differences in dosimeter response, calibration, and dosimetry practices. Cumulative organ absorbed dose was calculated by summing estimates of annual adjusted dose and applying sex-specific organ dose conversion coefficients published by the International Commission on Radiological Protection [25]. Consistent with previous studies [1, 11, 12], organ doses were calculated for the lung, active red bone marrow (RBM) for analyses of lymphohematopoietic cancers, and colon for analysis of all solid cancers combined (with and without lung cancer). To assess neutron exposures, available dosimetry records were used to classify whether a worker had a positive recorded neutron dose, and, if so, whether their recorded neutron dose ever exceeded 10% of their total external radiation dose of record [9, 18]. Consistent with main analyses in previous studies [1, 11, 12], no adjustment was made for potential internal exposures from incorporated radionuclides.

## Statistical methods

Associations between cumulative exposure and outcomes were modelled using Poisson regression, controlling for confounding via background stratification [26]. Model specifications used in the main analyses of previous studies were replicated in this study for comparisons [1, 11, 12], All models controlled for country, attained age (in 5-year intervals), sex, and year of birth (in 10-year intervals). Solid cancer outcomes additionally controlled for socioeconomic status (five categories, based on job title), duration of employment or radiation work (in 10-year intervals), and neutron monitoring status (a time-dependent categorical variable as previously described).

The dose-response association was modelled using a linear relative rate function as per previous analyses, with the measure of association expressed as ERR Gy^–1^. We noted considerable support for the selected model for most outcomes, although sensitivity analysis revealed evidence of modest downward curvature for lung cancer [1, 11, 12]. Given collinearity of temporal modifiers (i.e., AA, AE, and TSE), each was examined separately. Consistent with previous analyses, time windows of exposure were used, which account for exposures that have occurred at multiple points in the past. TSE was examined in time windows of exposure (10–<20, 20–<30, 30–<40, 40+ years) in analyses of solid cancers and lymphomas and (2–<10, 10–<20, 20–<30, 30–<40, 40+ years) in analyses of leukemias and MM. Window widths were chosen to maximize dose information supporting ERR Gy^–1^ estimation. Exposure windows were similarly defined for AE (< 35, 35–<50, 50+ years). Formally, for given *n* exposure windows and *k* levels of confounders, the death rate, I_*k*_, is modelled as:

$${I_k} = {\rm{ }}exp({a_k})\left( {1{\rm{ }} + {\beta _1}{Z_1} + {\beta _2}{Z_2} + {\beta _3}{Z_3} + {\rm{ }} \ldots {\rm{ }} + {\beta _n}{Z_n}} \right)$$

where Z_i_ is the cumulative dose in window *i* and *β*_*i*_ is the ERR Gy^–1^ for the *i*th window and exp(*α*_*k*_) is the baseline rate for *k* strata. Note that in a model in which *n* = 3, Z_1_ + Z_2_ + Z_3_ equals the total lagged cumulative dose, thus restricting *β*_1_ = *β*_2_ = *β*_3_ is equivalent to a standard lagged analysis of a cumulative dose-cancer mortality association. As a result, a likelihood ratio test (LRT) was used to assess heterogeneity in the ERR Gy^-1^ by TSE or AE.

Modification by AA was modelled in three windows (< 60, 60–<80 and 80 + years old) as:

$${I_k} = {\rm{ }}exp({\alpha _k})\left( {1{\rm{ }} + {\beta _1}{Z_1} + {\beta _2}{Z_2} + {\beta _3}{Z_3} + {\rm{ }} \ldots {\rm{ }} + {\beta _n}{Z_n}} \right)$$

Where I_< 60,_ for example, is an indicator variable (taking on value 1 or 0) indicating if the attained age of the *k*th strata is < 60 and Z is the total lagged cumulative dose.

A 10-year exposure lag for solid cancers and lymphomas and a 2-year lag for leukemias were selected a priori, as in previous analyses [1, 11, 12]. For MM, where a 10-year lag was used previously, a 2-year lag was selected based on goodness of fit in previous sensitivity analysis [12] to increase informativeness of TSE analysis.

Previous analyses examined the effects of restricting data pre- and post-1958 hires, and pre- and post-1965 hires [1, 12]. In solid cancer analyses, the ERR Gy^–1^ was greater in both the 1958+ and 1965+ subcohorts compared with the full cohort. To further investigate effects by hire period, the current study conducted similar sensitivity analyses applied to all outcomes; however, only the 1958 cutpoint was used given that the hire period effect for solid cancers was reasonably similar for both cutpoints [1].

*Post hoc* analyses were performed based on findings from planned analyses for both full and 1958 + cohorts. Estimates of ERR Gy^–1^ were reported with 90% Wald-based CIs. Some estimates were below the boundary of the relative rate because of linear extrapolation to 1 Gy. These estimates were reported without censor for completeness.

## Results

The cohort is mostly male (87%), with 103,553 total deaths (33.4%), including 28,089 solid cancers deaths, 8,266 lung cancer deaths, and 771 non-CLL leukemia deaths. The average age at end of follow-up was 65.9 years and ranged between 62.5 years in the UK to 71.4 years in the US. The average duration of follow-up was 34.6 years, ranging from 31.6 years among UK workers to 39.3 years for US workers. The radiation dose distribution was right-skewed, with average colon dose of 17.7 mGy (Table 1).

**Table 1:** Characteristics of the INWORKS cohort

Characteristic	France	UK	US	INWORKS
Calendar years of follow-up	1968–2014	1955–2012	1944–2016	1944–2016
Workers	60,697	147,872	101,363	309,932
Male	52,895	134,768	81,824	269,487
Female	7,802	13,104	19,539	40,445
Person-years (millions)	2.08	4.67	3.98	10.72
Mean duration of follow-up (years)	34.2	31.6	39.3	34.6
Mean age at end of follow-up (years)	64.8	62.5	71.4	65.9
Mean cumulative absorbed colon dose (mGy)	12.9	20.19	16.8	17.7
All deaths (%)	12,270 (20.2)	39,933 (27.0)	51,350 (50.7)	103,553 (33.4)
all solid cancers	4,446	11,574	12,069	28,089
lung, trachea, and bronchus	1,129	3,266	3,871	8,266
leukemia excl. CLL	122	264	385	771
CML	21	46	55	122
AML	54	160	221	435
MDS	19	34	110	163
NHL	160	387	599	1,146
MM	74	186	267	527

Abbreviations: AML, acute myeloid leukemia; CLL, chronic lymphocytic leukemia; CML, chronic myeloid leukemia; ICD 10, International Classification of Diseases, 10th revision; INWORKS, International Nuclear Workers Study; MDS, myelodysplastic syndrome; MM, multiple myeloma; NHL, non-Hodgkin lymphoma UK United Kingdom, US, United States of America

Statistically significant temporal modification of the ERR Gy^–1^ by TSE, AE, or AA was not observed in main or sensitivity analyses (Tables 2, 3 and 4, Online Resource Tables S2–S10). The results for each modifier, in main analyses first then followed by subcohort sensitivity analyses, are described below. Figures combining results from the full and restricted cohorts are provided in the online supplement (Online Resource Figures S1–S3).

**Table 2:** Linear ERR per gy by time since exposure (TSE).^1^

Outcome	ERR per Gy (90% CI) by time since exposure (years)					*P* ^2^
	2−<10	10−<20	20−<30	30−<40	40+	
solid cancers	NA	0.69 (–0.06, 1.43)	0.69 (0.03, 1.36)	0.52 (–0.12, 1.16)	0.00 (–0.59, 0.59)	0.479
solid cancers excl. lung	NA	0.77 (–0.15, 1.68)	0.35 (–0.47, 1.16)	0.57 (–0.19, 1.33)	0.04 (–0.64, 0.71)	0.738
lung cancer	NA	0.59 (–0.72, 1.90)	1.30 (0.09, 2.51)	0.57 (–0.64, 1.79)	–0.14 (–1.39, 1.12)	0.594
leukemias excl. CLL	–0.55 (–6.57, 5.46)	–0.84 (–6.25, 4.58)	7.29 (1.00, 13.6)	3.04 (–2.23, 8.32)	1.37 (–2.91, 5.65)	0.460
CML	11.5 (–10.5, 33.4)	–0.99 (–30.6, 28.6)	31.3 (1.50, 61.1)	12.4 (–18.4, 43.2)	0.68 (–20.0, 21.4)	0.409
AML	–0.41 (–8.99, 8.17)	–0.92 (–11.7, 9.89)	1.12 (–4.68, 6.92)	1.03 (–3.97, 6.04)	0.34 (–4.43, 5.12)	0.792
AML + MDS	–0.70 − 8.76, 7.35)	–0.91 (–10.2, 8.37)	1.92 (–3.61, 7.45)	1.68 (–3.02, 6.39)	2.66 (–1.12, 6.45)	0.647
MM	2.79 (–11.4, 17.0)	4.59 (–4.68, 13.9)	2.30 (–3.46, 8.06)	–0.62 (–4.82, 3.58)	0.71 (–3.93, 5.35)	0.756
NHL	NA	–0.92 (–4.69, 2.85)	0.36 (–2.86, 3.59)	0.60 (–2.79, 3.99)	0.57 (–2.76, 3.90)	0.773

^1^ Estimates below the boundary (i.e., ERR < − 1) that result from linear extrapolation are reported without censor

^2^ Test of the homogeneity of windows, where *P* is the p-value for the reported likelihood ratio test statistic and is evaluated under a Chi-square distribution with *k*-1 degrees of freedom for a model with *k* dose parameters

Abbreviations: CI, confidence interval; CLL, chronic lymphocytic leukemia; CML, chronic myeloid leukemia; ERR, excess relative rate; MDS, myelodysplastic syndrome; MM, multiple myeloma; NA, not applicable; NC, not calculable; NHL, non-Hodgkin lymphoma

### Time since exposure (TSE)

For TSE, the estimated ERR Gy^–1^ for solid cancers was 0.69 for TSE 10–<20 years and 20–<30 years, slightly smaller (ERR Gy^–1^ 0.52) for TSE 30–<40 years, and null (ERR Gy^–1^ 0.00) for TSE 40+ years, although differences were not significant (*P* = 0.479) (Table 2). The pattern was similar for solid cancers excluding lung cancer, although estimates varied less (*P* = 0.738). The lung cancer ERR Gy^–1^ peaked at TSE 20–<30 years, diminished but remaining positive for TSE 30–<40 years, and became negative for TSE 40+ years, with wide confidence intervals on the estimates. There was more variation in ERR Gy^–1^ with TSE for lymphohematopoietic cancers compared with solid cancers (Table 2). The non-CLL leukemia ERR Gy^–1^ was greatest at TSE 20–<30 years (ERR Gy^–1^ = 7.29), then declined thereafter, although remained positive at TSE 40+ years (ERR Gy^–1^ = 1.37). A positive ERR Gy^–1^ for non-CLL leukemia and AML was not observed prior to 20 years TSE. The temporal pattern in ERR Gy^–1^ for CML appeared bimodal, with peaks occurring at 2–<10 years and at 20–<30 years. Like CML, the ERR Gy^–1^ estimate for MM was positive in the 2–<10-year window suggesting a short exposure lag; however, estimates were largely imprecise. Positive associations for NHL were observed beginning at TSE 20–<30 years and persisted in remaining windows, suggesting a pattern of late onset NHL, although estimates were imprecise.

## Age at exposure (AE)

The solid cancer ERR Gy^–1^ was closer to null for AE < 35 years than for AE 35–<50 years and 50+ years (Table 3). A similar patterns was observed for solid cancers excluding lung cancer, but with less variability between estimates. The attention at AE < 35 years was greatest for lung cancer. Positive associations were evident in all AE categories for non-CLL leukemia, with estimates greatest in magnitude at AE > 35 years. This pattern was driven by increased CML risk at later AE. In contrast, the estimate for AML mortality was greatest at AE < 35 years, although estimates were imprecise. MM mortality showed the greatest heterogeneity by AE (*P* = 0.174) and the ERR Gy^–1^ estimate for MM mortality was greatest in magnitude at AE 50+. In contrast, there was little evidence of modification of NHL by AE (*P* = 0.840), with the greatest ERR Gy^–1^ at AE < 35 years.

**Table 3:** Linear ERR per gy by age at exposure (AE).^1^

Outcome	Lag (years)	ERR per Gy (90% CI) by age at exposure (years)			*P* ^2^
		< 35	35−<50	50+	
solid cancers	10	0.01 (–0.59, 0.61)	0.68 (0.23, 1.12)	0.56 (–0.05, 1.17)	0.365
solid cancers excl. lung	10	0.15 (–0.55, 0.84)	0.46 (–0.06, 0.98)	0.57 (–0.15, 1.30)	0.753
lung cancer	10	–0.35 (–1.52, 0.82)	1.22 (0.35, 2.10)	0.55 (–0.58, 1.69)	0.282
leukemias excl. CLL	2	0.94 (–3.18, 5.06)	3.13 (0.07, 6.18)	3.47 (–0.66, 7.60)	0.757
CML	2	–1.46 (–13.5, 10.6)	13.4 (1.60, 25.1)	10.7 (–4.08, 25.4)	0.394
AML	2	1.97 (–3.03, 6.97)	0.05 (–3.42, 3.53)	0.71 (–3.66, 5.07)	0.897
AML + MDS	2	2.83 (–1.74, 7.40)	1.36 (–2.07, 4.80)	0.86 (–3.11, 4.83)	0.846
MM	2	3.22 (–1.83, 8.27)	–0.92 (–3.66, 1.82)	6.40 (0.56, 12.2)	0.174
NHL	10	1.06 (–1.94, 4.06)	–0.13 (–2.20, 1.95)	–0.15 (–2.87, 2.58)	0.840

^1^ Estimates below the boundary (i.e., ERR < − 1) that result from linear extrapolation are reported without censor

^2^ Test of the homogeneity of windows, where *P* is the p-value for the reported likelihood ratio test statistic and is evaluated under a Chi-square distribution with 2 degrees of freedom

Abbreviations: AML, acute myeloid leukemia; CI, confidence interval; CLL, chronic lymphocytic leukemia; CML, chronic myeloid leukemia; ERR, excess relative rate; MM, multiple myeloma; NHL, non-Hodgkin lymphoma

**Table 4:** Linear ERR per gy by attained age (AA).^1^

Outcome	Lag (years)	ERR per Gy (90% CI) by attained age (years)			*P* ^2^
		< 60	60−<80	80+	
solid cancers	10	0.42 (–0.44, 1.29)	0.42 (0.12, 0.72)	0.71 (0.21, 1.22)	0.691
solid cancers excl. lung	10	0.82 (–0.25, 1.89)	0.23 (–0.12, 0.58)	0.69 (0.14, 1.25)	0.398
lung cancer	10	–0.68 (–2.19, 0.83)	0.81 (0.23, 1.39)	0.81 (–0.39, 2.01)	0.375
leukemias excl. CLL	2	1.32 (–2.80, 5.44)	1.74 (–0.27, 3.74)	6.07 (1.57, 10.6)	0.240
CML	2	8.57 (–4.35, 21.5)	5.62 (–0.94, 12.2)	40.5 (–7.22, 88.2)	0.131
AML	2	0.37 (–4.58, 5.32)	–0.82 (–2.99, 1.35)	1.65 (–1.73, 5.04)	0.292
AML + MDS	2	2.37 (–3.92, 8.67)	–0.38 (–2.25, 1.49)	4.20 ( 0.73, 7.67)	0.115
MM	2	4.28 (–2.65, 11.2)	1.69 (–0.45, 3.84)	0.35 (–2.51, 3.22)	0.624
NHL	10	2.90 (–2.02, 7.82)	–0.25 (–1.41, 0.92)	0.42 (–1.69, 2.52)	0.498

^1^ Estimates below the boundary (i.e., ERR < − 1) that result from linear extrapolation are reported without censor

^2^ Test of the homogeneity of windows, where *P* is the p-value for the reported likelihood ratio test statistic and is evaluated under a Chi-square distribution with 2 degrees of freedom

Abbreviations: AML, acute myeloid leukemia; CI, confidence interval; CLL, chronic lymphocytic leukemia; CML, chronic myeloid leukemia; ERR, excess relative rate; MDS, myelodysplastic syndrome; MM, multiple myeloma; NHL, non-Hodgkin lymphoma

## Attained age

Although estimates were imprecise, there was greater heterogeneity in ERR Gy^–1^ by AA for leukemias than for solid cancers, MM or NHL. Estimates were generally greater in magnitude at AA 80+ years relative to other age categories for all outcomes except for MM and NHL, where estimates appeared greatest in the youngest age group (age < 60 years).

### Sensitivity analysis

The average attained ages were 75.7 years and 62.9 years, for hire years < 1958 (*n* = 71, 293) and 1958+ (*n* = 238,639), respectively. The average follow-up was 40.0 and 33.0 years, for hire years < 1958 and 1958+, respectively. The age at cohort entry (a proxy for first exposure) was greater among those hired prior to 1958 (mean age 35.7 years) compared with those hired 1958+ (mean age 29.9 years). The average radiation colon dose was 30.3 mGy among the < 1958 hires compared with 13.9 mGy for workers hired in 1958 and later. (Online Resource Table S2).

Compared with main analyses, there was generally less variation in ERR Gy^–1^ by TSE, but substantially decreased estimate precision in subcohort analyses, (Online Resource Tables S3 and S4). Solid cancer estimates in the 1958+ cohort were generally greater in magnitude than that in the < 1958 subcohort. This difference was greatest for lung cancer, where the ERR Gy^–1^ estimate for the < 1958 subcohort was negative at TSE 30 years of greater. The 1958+ cohort yielded positive ERRs in all TSE categories for solid cancers, solid cancer excluding lung cancer, and lung cancer. However, the variation in ERR Gy^–1^ by hire period persisted in *post hoc* solid cancer analyses restricting data to TSE 10–<40 years, suggesting that the difference in ERR Gy^–1^ for solid cancer by hire period was not wholly attributable to the greater length of follow-up among earlier hires (Online Resource Table S5). In contrast to the < 1958 subcohort, there was no evidence of early onset MM in the 1958+ subcohort (Online Resource Table S3 and S4).

The ERR Gy^–1^ estimates for solid cancers and solid cancers excluding lung were closer to null for AE < 35 years than for AE 35–<50 years and 50+ years in both subcohorts (Online Resource Tables S6 and S7). However, the ERR Gy^–1^ estimate for lung cancer was negative at AE 50+ years after restricting to 1958+ hires (Online Resource Table S6) and greatest at AE 50 + among < 1958 hires (Online Resource Table S7). The solid cancer pattern was further elucidated in two-window *post hoc* analyses (Online Resource Table S8), which also shows the largest difference in ERR Gy^–1^ by AE in the full cohort, with nearly all the risk apportioned to AE 35+ years. There were positive associations between radiation dose accrued at AE > 35 years for non-CLL leukemia in full and restricted cohorts (Table 3, Online Resource Tables S6 and S7). The MM ERR Gy^–1^ was greatest in magnitude at AE 50+, with the largest estimate among workers hired prior to 1958 (Online Resource Table S7).

In the 1958+ subcohort, the ERR Gy^–1^ estimate was greatest in magnitude at AA 80+ years for all outcomes. This pattern persisted in the < 1958 subcohort except for lung cancer, MM, and NHL. The lung cancer ERR Gy^–1^ was greatest at AA 60–<80 years, although remained positive in the 80+ year category. The MM ERR Gy^–1^ was greatest at AA 60–<80 years and NHL estimate was greatest at AA < 60 years.

## Discussion

We examined potential temporal modifiers of the ERR Gy^–1^ using updated INWORKS data on mortality from all solid cancers, solid cancers excluding lung cancer, lung cancer, leukemias, myelomas, and NHL. There was no statistically significant effect measure modification for any cancer examined, but we did observe interesting patterns, such as a peak and subsequent decline in the CML ERR Gy^–1^ with TSE, elevated ERR Gy^–1^ for leukemia 20 or more years after exposure, and late-onset NHL.

TSE patterns were largely consistent with those in previous analyses except for CML, where there was early evidence of strong and temporally complex modification of the CML ERR Gy^–1^ by TSE (*P* = 0.021) [4]. The previously observed bimodal pattern in ERR Gy^–1^ with TSE in CML persisted in the current study, although ERR Gy^–1^ variation was less pronounced. TSE analyses of leukemia excluding CLL, and AML, found positive ERR Gy^–1^ estimates first observed 20–<30 years after exposure. While risk models for leukemia typically assume a minimum 2-year lag, these results suggest a longer lag for these associations among these nuclear workers.

Except for MM and NHL, we observed a general pattern of increasing ERR Gy^-1^ estimates at AE 35–<50 years compared with AE < 35 years. There is little evidence of association for all solid cancers, solid cancers excluding lung, or lung cancer with AE < 35 years. For MM, the ERR Gy^-1^ was greatest at AE 50 years or later, while for NHL, the estimate was positive only in the youngest AE category. Some previous studies have found positive effect modification by age at exposure among nuclear workers [27–29], while others have not [21–24, 30]. Reasons for changes in radiosensitivity with age among adults are not clear; however, age-related physiological changes or tumor behaviors have been offered as possible explanations [31–33].

Our study is first to examine AA effect modification in INWORKS. Estimates of ERR Gy^–1^ in AA categories appeared largely comparable across attained age groups for most outcomes. With few exceptions, there was a general pattern of increased ERR Gy^–1^ at oldest attained ages compared with younger age groups. The reasons for this increase are not clear; however, estimates of association at the oldest attained ages tended to be imprecise.

### Comparisons with separate analyses of INWORKS subcohorts

Time since first exposure, age at first exposure, and attained age were examined in studies of cancer incidence in UK NRRW workers followed through 2011 [23, 24]. There was significant modification (*P* = 0.007) of ERR Sv^–1^ for all solid cancers by age at first exposure, with workers initially exposed at age 30+ years (ERR Sv^–1^ = 0.39; 95% CI: 0.14, 0.67) having greater risk compared to those aged < 30 years (ERR Sv^–1^ = − 0.07; 95% CI: − 0.32, 0.214) [24]. There was no evidence of modification by AA (*P* = 0.49) or time since first exposure (test results not reported). Similarly, temporal variation in the ERR Sv^–1^ was not found in analyses examining the incidence of solid cancers excluding lung cancer, lung cancer, MM, or lymphoma [23, 24]. In a separate study, Gillies et al. found little evidence of temporal variation in the ERR Sv^–1^ by AA (*P* = 0.10), AE (*P* = 0.24) or TSE (*P* = 0.37) for non-CLL leukemia mortality and incidence in UK males [30]. Despite differences in definitions of temporal modifiers, the patterns observed in the NRRW studies were reasonably consistent with those found in INWORKS.

In analyses of the association between equivalent dose and solid cancers, there was little evidence of strong variation in the ERR Sv^–1^ by AE or TSE for solid cancers combined or lung cancer in US nuclear workers followed through 2016 [21]. However, an earlier study of the US workers followed through 2005 found a significantly positive interaction between radiation and AA (*P* = 0.011) for nonsmoking-related cancers combined [34]. That study also found significant heterogeneity in ERR Gy^–1^ for MM with TSE (*P* = 0.047), with the largest ERR Gy^–1^ in the 10–<20-year category, which is also observed in INWORKS full cohort analyses.

In the most recent study of the French nuclear workers followed through 2014, there was absence of substantial effect modification of the solid cancer mortality ERR Gy^–1^ by AA and AE [22]. In contrast, including AA as a modifier of ERR Gy^–1^ for non-CLL leukemia improved model fit (as determined by Akaike information criteria), with ERR Gy^–1^ increasing with AA. This pattern is also evident in INWORKS, albeit not statistically significant.

### Comparisons with the LSS

The Life Span Study (LSS) of atomic bomb survivors is well-positioned to examine time-related modifiers of ERR Gy^–1^ following acute radiation exposure given that the cohort includes persons of all ages who were exposed at a single point in time. There is strong evidence of temporal modification of the ERR Gy^–1^ for solid cancer mortality and incidence in the LSS full cohort [35–37]. In a recent mortality study, the ERR Gy^–1^ for all solid cancers declined 29% (95% CI: − 41%, − 17%) per 10-year increase in AE and in proportion to the − 0.86 (95% CI: − 1.60, − 0.06) power of AA [35]. Similar models were fit for LSS lung cancer mortality; however, the modification was more subtle, with decreases of 7% (95% CI: − 35%, 29%) per 10-year increase in AE and in proportion to − 0.04 (–2.2, 2.6) power of AA. In contrast, our study of working adults provided little evidence of decline in the solid cancer ERR Gy^–1^ with AE or AA, although there was attenuation at TSE 40 years for all outcomes except MM and NHL. In a study comparing mortality in INWORKS and the LSS, data were restricted to populations that were similar in age at exposure (20–60 years), birth cohort, and 5-year exposure lag [5]. That study found only modest evidence of modification by AA in the LSS, and no evidence of modification by AE after restriction to adult working ages at exposure. Exposure-time differences, dissimilarities in age ranges (all ages vs. adults) and other population characteristics, as well as limitations in statistical power and analytic methods, might explain some discordance in solid cancer findings between INWORK and the LSS.

There was variation with TSE in the leukemia mortality ERR Gy^–1^ in the LSS cohort followed through 2000 [38]. The empirically modeled dose-response suggested a bimodal pattern that included a period of early elevation in the ERR Gy^-1^ followed by a later increase decades after exposure. Also, under the fitted LSS model, the leukemia ERR Gy^–1^ decreased with increasing AE through age 30 years, suggesting little evidence of AE modification among adults. These general patterns were consistent with our findings, although early onset was substantially attenuated in LSS adults aged 30 years or more. The authors speculated that the evidence of short latency may represent effects among a susceptible population via immediate clonal expansion of preleukemic cells. In turn, the late onset was believed to be caused by different late-acting mechanisms of induction and malignant transformation. We noted that the empirical model described for mortality was a poorer fit to leukemia incidence data, where the preferred model was linear-quadratic in dose (upward curvature) with decreases in the ERR Gy^–1^ with increasing AA (proportional to the power −1.09) and TSE (proportional to the power − 0.81) [39]. Although the ERR Gy^–1^ decreased with increasing TSE, that study reported significant leukemia risk that persisted over the entire follow-up period.

Richardson and colleagues examined TSE modification of the association between radiation and lymphoma mortality in a subgroup of LSS males aged 15–64 years at the time of the bombings [40]. That study found no evidence of association 5–35 years after exposure (ERR Sv^–1^ = 0.03, 90% CI: ND, 1.15); however, a modest but significant association was observed at TSE 36 + years (ERR Sv^–1^ = 1.93, 90% CI: 0.48, 4.66). In contrast, significant modification of the NHL ERR Gy^–1^ was not observed in our analysis, with all estimates indistinguishable from the null. Still, the NHL ERR Gy^–1^ appeared greatest at TSE 30 years and later, which is consistent with the findings from the LSS and previous INWORKS reports, suggesting extended latency and persistence of NHL from radiation exposure. This finding is consistent with a positive NHL ERR Gy^–1^ found only at youngest AE, which allowed for sufficient time to elapse to observe a dose-response association. There was evidence of a radiation effect in men, but not women in the recent LSS study of NHL incidence [39]. In contrast to the mortality study, that study found the effect among men was best described by a linear model allowing the ERR Gy^–1^ to decline with age.

There were no investigations of temporal modifiers for LSS MM mortality. In a study of MM incidence, analyses of AA and AE did not reveal significant modification, and the ERR Gy^–1^ estimate from a linear dose-response model was modest and not significant (ERR Gy^–1^ = 0.38; 95% CI: − 0.23, 1.36) [39]. The absence of temporal analyses of MM mortality is likely due to limitations from small numbers and modest radiation effects.

### Modification by date of Hire

INWORKS has reported greater estimates of the solid cancer ERR Gy^–1^ among persons hired in 1958 or later (ERR Gy^–1^ = 1.22; 90% CI 0.74, 1.72) and in 1965 and later (ERR Gy^–1^ = 1.44; 90% CI 0.65, 2.32) compared with workers hired prior to 1958 (ERR Gy^–1^ = 0.20; 90% CI − 0.07, 0.49) [1]. In contrast, there was little evidence of a ‘hire date effect’ in the most recent analysis of non-CLL leukemia mortality [12]. Causes have not been elucidated, which prompted commentary calling for further investigation [41]. We conducted additional analyses of temporal factors by hire year, which also show a pattern of smaller ERR Gy^–1^ among workers hired earlier compared with later hires. In INWORKS, those hired prior to 1958, on average, have greater length of follow-up, older attained age, and higher total cumulative dose compared to those hired after (Online Resource Table S2). Workers hired early generally contribute more to later TSE, AE, and AA simply because they are followed longer; however, the variation in ERR Gy^–1^ by hire period appeared unrelated to length of follow-up in our analyses.

Solid cancer results appear largely driven by lung cancer, an outcome that is associated with several modifiable factors (e.g., smoking, lifestyle, other occupational and environmental exposures) that may change over time; therefore, hire year may be a proxy for unmeasured time-varying factors. There is no evidence of strong variation in ERR Gy^–1^ by hire period for non-CLL leukemia ERR Gy^–1^ [12]. In general, non-CLL leukemia exhibits a stronger association with radiation but weaker association with smoking and lifestyle factors when compared with lung cancer, suggesting less potential for strong time-varying confounding by smoking and lifestyle factors for leukemia than lung cancer.

The magnitude of the variation in ERR Gy^–1^ for solid cancer by hire period differs by country. In previous analysis of US workers, the estimate of solid cancer mortality in the full cohort (ERR Sv^–1^ = 0.19; 95% CI: − 0.10, 0.52) was markedly less than that following restriction to workers hired in 1960 or later (ERR Sv^–1^ = 2.23; 95% CI: 1.13, 3.49) [21]. The pattern was also evident, albeit attenuated and not significant (*P* = 0.20), in a reanalysis of solid cancer incidence among UK NRRW workers, where the ERR Sv^–1^ among workers hired in 1960 and later (ERR Sv^–1^ = 0.39; 95% CI: 0.04, 0.76) was greater than that for workers hired prior to 1960 (ERR Sv^–1^ = 0.14; 95% CI: − 0.08, 0.38) [42]. The difference was largely attributable to lung cancer incidence, where the ERR Sv^–1^ for those hired 1960+ (ERR Sv^–1^ = 0.92; 95% CI: − 0.01, 2.00) was much greater than that for earlier hires (ERR Sv^–1^ = − 0.01; 95% CI: − 0.39, 0.52). Observations in the UK NRRW cohort are limited due to small numbers of early hires. The recent study of French nuclear workers, who contribute the fewest early hires to INWORKS, reported little change in the solid cancer ERR Gy^–1^ after restricting to those hired after 1956 [22]. Although there are inconsistencies among these studies that are caused, in part, by different analytic approaches, a pattern emerges where the variation in ERR Gy^–1^ for solid cancer increases with the proportion of early hires. Within the < 1958 subcohort, US workers contributed 59% of the solid cancer deaths and 62% of the person-years at risk (Online Resource Table S7). Wartime nuclear work is unique to US workers who developed the first atomic bombs in the 1940s at Hanford and ORNL, while UK and French nuclear workers started operations later, with follow-up for UK workers beginning in 1955 and for French workers in 1968. Confounding or selection associated with early worker characteristics (e.g., lifestyle, health, environmental exposures) and war era conditions might account for observed differences in solid cancer ERR Gy^–1^ by hire period and should be investigated in future analyses.

Other potential sources of bias include time-varying dosimetry error [43], although this seems unlikely to fully explain the effect in solid cancers, but not in non-CLL leukemia, both using the same dosimetry records. Nevertheless, the current analysis lacks sufficient study data to confirm or eliminate measurement error as a cause. It is also recognized that there is evidence of modest downward curvature of the dose-response for solid cancers, primarily for lung cancer, but not for non-CLL leukemia. Additional investigation into the potential relationship between the shape of the solid cancer dose-response, particularly that for lung cancer, and the variation in ERR Gy^–1^ by hire period is needed.

## Conclusion

We examined TSE, AE, and AA and found little evidence of strong effect modification of ERR Gy^–1^ by any temporal factor. Temporal patterns of variation in ERR Gy^–1^ were consistent with previous studies of INWORKS and country-specific analyses; however, poor statistical power remains as a major limitation and was a consideration for not conducting joint temporal analyses as done previously [4]. Considering estimate imprecision, our findings were reasonably compatible with those observed in the LSS, especially when restricting LSS exposures to working ages. There was continued evidence of meaningful radiation effects decades after exposure, which is an important consideration in estimating lifetime risks. Variation in the solid cancer ERR Gy^–1^ by hire period could, in theory, be due to differences in the distribution of effect measure modifiers by calendar period of hire. However, our analyses suggest that this is not the case for the temporal modifiers we examined (TSE, AE, AA). Further investigation that is beyond the scope of the current work is needed to explore other potential causes.

## Electronic supplementary material

Below is the link to the electronic supplementary material.

Supplementary Material 1

## Declarations

### Disclaimer

The findings and conclusions in this report are those of the authors and do not necessarily represent the views of the National Institute for Occupational Safety and Health. Where authors are identified as personnel of the International Agency for Research on Cancer/World Health Organization, the authors alone are responsible for the views expressed in this article and they do not necessarily represent the decisions, policy, or views of the International Agency for Research on Cancer/World Health Organization.
