# Supplementary material for: Updated findings on temporal variation in radiation-effects on cancer mortality in an international cohort of nuclear workers (INWORKS)
Source: Eur J Epidemiol. 2024 Nov 22;39(11):1277–86. doi: 10.1007/s10654-024-01178-6 (PMC11646264; doi:10.1007/s10654-024-01178-6)
Supplement: Supplementary file 1 — Supplementary Material 1 [file 10654_2024_1178_MOESM1_ESM.docx]

ONLINE RESOURCE SUPPLEMENTAL INFORMATION FOR:

Updated findings on temporal variation in radiation-effects on cancer mortality in an international cohort of nuclear workers (INWORKS), Euro J Epidemiol.

Robert D. Daniels^*^, Stephen J. Bertke, Kaitlin Kelly-Reif, David B. Richardson, Richard Haylock, Dominique Laurier, Klervi Leuraud, Monika Moissonnier, Isabelle Thierry-Chef, Ausrele Kesminiene, and Mary K. Schubauer-Berigan

^*^Corresponding author: 1090 Tusculum Avenue, Mailstop 12, Cincinnati, OH 45226. Email: rtd2@cdc.gov; Phone: (513) 533-8329; Cell (513) 594-2764; ORCID: 0000-0002-3915-7991.

Contents

[Table S1. Outcome categories and associated codes in revisions 6–10 of the International Classification of Diseases (ICD, WHO) used for the analysis of temporal effect modification in INWORKS. 2](file:///C:\Users\is10747\AppData\Local\Temp\a3ba96fb-8d81-4dff-bb44-f6352b56f198_EJEP-D-24-00638.zip.198\TEMPORAL%20Supplement%2010052024.docx#_Toc170712185)

[Table S2. Characteristics of the INWORKS cohort stratified by hire date. 3](file:///C:\Users\is10747\AppData\Local\Temp\a3ba96fb-8d81-4dff-bb44-f6352b56f198_EJEP-D-24-00638.zip.198\TEMPORAL%20Supplement%2010052024.docx#_Toc170712186)

[Table S3. Linear ERR per Gy by time since exposure (TSE) in INWORKS restricted to workers hired in 1958 and later. 4](file:///C:\Users\is10747\AppData\Local\Temp\a3ba96fb-8d81-4dff-bb44-f6352b56f198_EJEP-D-24-00638.zip.198\TEMPORAL%20Supplement%2010052024.docx#_Toc170712187)

[Table S4. Linear ERR per Gy by time since exposure (TSE) in INWORKS restricted to workers hired prior to 1958. 5](file:///C:\Users\is10747\AppData\Local\Temp\a3ba96fb-8d81-4dff-bb44-f6352b56f198_EJEP-D-24-00638.zip.198\TEMPORAL%20Supplement%2010052024.docx#_Toc170712188)

[Table S5. Post hoc solid cancer analyses: linear ERR per Gy (90% CI) by time since exposure (TSE) in INWORKS. 6](file:///C:\Users\is10747\AppData\Local\Temp\a3ba96fb-8d81-4dff-bb44-f6352b56f198_EJEP-D-24-00638.zip.198\TEMPORAL%20Supplement%2010052024.docx#_Toc170712189)

[Table S6. Linear ERR per Gy by age at exposure (AE) in INWORKS restricted to workers hired in 1958 and later. 7](file:///C:\Users\is10747\AppData\Local\Temp\a3ba96fb-8d81-4dff-bb44-f6352b56f198_EJEP-D-24-00638.zip.198\TEMPORAL%20Supplement%2010052024.docx#_Toc170712190)

[Table S7. Linear ERR per Gy by age at exposure (AE) in INWORKS restricted to workers hired prior to 1958. 8](file:///C:\Users\is10747\AppData\Local\Temp\a3ba96fb-8d81-4dff-bb44-f6352b56f198_EJEP-D-24-00638.zip.198\TEMPORAL%20Supplement%2010052024.docx#_Toc170712191)

[Table S8. Post hoc solid cancer analyses: linear ERR per Gy by age at exposure (AE) in INWORKS. 9](file:///C:\Users\is10747\AppData\Local\Temp\a3ba96fb-8d81-4dff-bb44-f6352b56f198_EJEP-D-24-00638.zip.198\TEMPORAL%20Supplement%2010052024.docx#_Toc170712192)

[Table S9. Linear ERR per Gy by attained age (AA) in INWORKS restricted to workers hired in 1958 and later. 10](file:///C:\Users\is10747\AppData\Local\Temp\a3ba96fb-8d81-4dff-bb44-f6352b56f198_EJEP-D-24-00638.zip.198\TEMPORAL%20Supplement%2010052024.docx#_Toc170712193)

[Table S10. Linear ERR per Gy by attained age (AA) in INWORKS restricted to workers hired prior to 1958. 11](file:///C:\Users\is10747\AppData\Local\Temp\a3ba96fb-8d81-4dff-bb44-f6352b56f198_EJEP-D-24-00638.zip.198\TEMPORAL%20Supplement%2010052024.docx#_Toc170712194)

[Figure S1. The ERR Gy–1 (filled circles) and associated 90% CIs (whiskers) for categories of time since exposure (TSE) for all solid cancers and leukemia excluding CLL. 12](file:///C:\Users\is10747\AppData\Local\Temp\a3ba96fb-8d81-4dff-bb44-f6352b56f198_EJEP-D-24-00638.zip.198\TEMPORAL%20Supplement%2010052024.docx#_Toc170712195)

[Figure S2. The ERR Gy–1 (filled circles) and associated 90% CIs (whiskers) for categories of age at exposure (AE) for all solid cancers and leukemia excluding CLL. 13](file:///C:\Users\is10747\AppData\Local\Temp\a3ba96fb-8d81-4dff-bb44-f6352b56f198_EJEP-D-24-00638.zip.198\TEMPORAL%20Supplement%2010052024.docx#_Toc170712196)

[Figure S3. The ERR Gy–1 (filled circles) and associated 90% CIs (whiskers) for categories of attained age for all solid cancers and leukemia excluding CLL. 14](file:///C:\Users\is10747\AppData\Local\Temp\a3ba96fb-8d81-4dff-bb44-f6352b56f198_EJEP-D-24-00638.zip.198\TEMPORAL%20Supplement%2010052024.docx#_Toc170712197)

# Table S1. Outcome categories and associated codes in revisions 6–10 of the International Classification of Diseases (ICD, WHO) used for the analysis of temporal effect modification in INWORKS.

| Outcome | ICD-6 | ICD-7 | ICD-8 | ICD-9 | ICD-10 |
| --- | --- | --- | --- | --- | --- |
| solid cancers^1^ | 140–199 | 140–199 | 140–199 | 140–199, except 176.5 (Kaposi’s sarcoma of lymph nodes) | C00–C80, C97, except C46.3 (Kaposi’s sarcoma of lymph nodes) |
| lung cancer | 162–163 | 162.0–162.1, 162.8, 163 (pleura excluded) | 162 | 162 | C33–C44 |
| leukemia excl. CLL | 204.1–204.3 | 204.1–204.3 | 204.0, 205.0–207.0, 207.2 | 204.0, 204.2–204.8, 205–208.0, 208.2–208.8 | C91.0, C91.2–C91.7, C92.0–C95.0, C95.2–C95.7 |
| CML | NA | NA | 205.1 | 205.1, 206.1 | C92.1, C93.1, C94.8 |
| AML | NA | NA | 205.0, 206.0 | 205.0, 205.3, 206.0, 207.0, 207.2 | C92.0, C92.3–C92.6, C93.0, C94.0, C94.2, C94.4, C94.5 |
| MDS | NA | NA | NA | NA | D46 |
| NHL | 200, 202, 205 | 200, 202, 205 | 200, 202, 275.5 | 200, 202, 273.3 | C82–C85, C88, C96 |
| MM | 203 | 203 | 203 | 203 | C90 |

^1^ In addition to all solid cancers, solid cancers excluding lung cancer was assessed separately but not explicitly shown in the table. For solid cancers excluding lung, the associated codes for lung cancer, as shown in the table above, were omitted.

Abbreviations: AML, acute myeloid leukemia; CLL, chronic lymphocytic leukemia; CML, chronic myeloid leukemia; MDS, myelodysplastic syndrome; MM, multiple myeloma; NA, not applicable; NHL, non-Hodgkin lymphoma.

# Table S2. Characteristics of the INWORKS cohort stratified by hire year (<1958, 1958+).

| Characteristic | France (1968–2014) | | | UK (1955–2012) | | | US (1944–2016) | | | INWORKS (1944–2016) | | |
| --- | --- | --- | --- | --- | --- | --- | --- | --- | --- | --- | --- | --- |
|  | <1958 | 1958+ | Full | <1958 | 1958+ | Full | <1958 | 1958+ | Full | <1958 | 1958+ | Full |
| workers | 5,156 | 55,541 | 60,697 | 24,449 | 123,423 | 147,872 | 41,688 | 59,675 | 101,363 | 71,293 | 238,639 | 309,932 |
| person-years (millions) | 0.19 | 1.89 | 2.08 | 0.89 | 3.78 | 4.67 | 1.78 | 2.20 | 3.98 | 2.86 | 7.87 | 10.72 |
| male | 0.16 | 1.64 | 1.8 | 0.83 | 3.44 | 4.27 | 1.47 | 1.70 | 3.19 | 2.46 | 6.78 | 9.72 |
| female | 0.031 | 0.25 | 0.28 | 0.05 | 0.34 | 0.40 | 0.31 | 0.50 | 0.81 | 0.39 | 1.09 | 1.48 |
| mean follow-up (years) | 36.4 | 34.0 | 34.2 | 36.2 | 30.6 | 31.6 | 42.7 | 36.9 | 39.3 | 40.0 | 33.0 | 34.6 |
| mean age at EOF (years) | 78.2 | 63.5 | 64.8 | 74.0 | 60.2 | 62.5 | 76.4 | 67.9 | 71.4 | 75.7 | 62.9 | 65.9 |
| mean colon dose (mGy) | 18.0 | 12.4 | 12.9 | 39.5 | 16.4 | 20.2 | 26.5 | 10.1 | 16.8 | 30.3 | 13.9 | 17.7 |
| solid cancer deaths | 1,055 | 3,391 | 4,446 | 4,353 | 7,221 | 11,574 | 7,813 | 4,256 | 12,069 | 13,221 | 14,868 | 28,089 |
| lung cancer deaths | 243 | 886 | 1,129 | 1,259 | 2,007 | 3,266 | 2,588 | 1,283 | 3,871 | 4,090 | 4,176 | 8,266 |
| leukemia, excl. CLL deaths | 22 | 100 | 122 | 105 | 159 | 264 | 239 | 146 | 385 | 366 | 405 | 771 |

Abbreviations: CLL, chronic lymphocytic leukemia; INWORKS, International Nuclear Workers Study; UK United Kingdom, US, United States of America.

# Table S3. Linear ERR per Gy by time since exposure (TSE) in INWORKS restricted to workers hired in 1958 and later.^1^

| Outcome | ERR per Gy (90% CI) by time since exposure (years) | | | | | *P* ^2^ |
| --- | --- | --- | --- | --- | --- | --- |
|  | 210 | 1020 | 2030 | 3040 | 40+ |  |
| solid cancers | NA | 1.82 (0.57, 3.07) | 0.58 (–0.50, 1.65) | 1.55 (0.45, 2.65) | 0.51 (–0.97, 1.99) | 0.629 |
| solid cancers excl. lung | NA | 2.23 (0.69, 3.77) | 0.53 (–0.76, 1.82) | 1.40 (0.11, 2.69) | 0.29 (–1.35, 1.93) | 0.511 |
| lung cancer | NA | 0.91 (–1.28, 3.09) | 0.56 (–1.42, 2.53) | 2.12 (–0.06, 4.30) | 1.37 (–2.01, 4.75) | 0.837 |
| leukemias excl. CLL | –1.10 (–17.8, 15.6) | –0.73 (–13.9, 12.5) | 5.03 (–3.56, 13.6) | 1.99 (–5.81, 9.79) | 2.48 (–5.21, 10.2) | 0.603 |
| CML | 28.9 (–23.0, 80.8) | –0.34 (–171, 170) | 34.9 (–21.1, 90.8) | –1.18 (–60.9, 58.6) | 25.8 (–31.1, 82.7) | 0.353 |
| AML | –1.10 (–93.6, 91.4) | –0.49 (–24.0, 23.0) | 1.45 (–8.76, 11.7) | 0.39 (–7.92, 8.69) | 0.67 (–6.55, 7.90) | 0.864 |
| AML+MDS | –1.10 (–84.2, 82.1) | –0.10 (–17.5, 17.3) | 2.28 (–6.33, 10.9) | 1.41 (–5.83, 8.66) | 1.24 (–5.09, 7.57) | 0.861 |
| MM | –0.81 (–23.4, 21.8) | –1.09 (–14.2, 12.0) | 0.73 (–6.34, 7.80) | 3.06 (–6.18, 12.3) | 2.48 (–10.1, 15.1) | 0.824 |
| NHL | NA | –1.10 (–6.59, 4.40) | 0.35 (–4.21, 4.91) | 0.87 (–4.48, 6.21) | 0.62 (–6.63, 7.86) | 0.803 |

^1^ Estimates below the boundary (i.e., ERR < –1) that result from linear extrapolation are reported without censor.

^2^ Test of the homogeneity of windows, where *P* is the p-value for the reported likelihood ratio test statistic and is evaluated under a Chi-square distribution with *k*-1 degrees of freedom for a model with *k* dose parameters.

Abbreviations: CI, confidence interval; CLL, chronic lymphocytic leukemia; CML, chronic myeloid leukemia; ERR, excess relative rate; MDS, myelodysplastic syndrome; MM, multiple myeloma; NA, not applicable; NHL, non-Hodgkin lymphoma.

# Table S4. Linear ERR per Gy by time since exposure (TSE) in INWORKS restricted to workers hired prior to 1958.^1^

| Outcome | ERR per Gy (90% CI) by time since exposure (years) | | | | | *P* ^2^ |
| --- | --- | --- | --- | --- | --- | --- |
|  | 210 | 1020 | 2030 | 3040 | 40+ |  |
| solid cancers | NA | –0.16 (–1.10, 0.77) | 0.86 (0.00, 1.73) | –0.25 (–1.04, 0.55) | 0.10 (–0.57, 0.78) | 0.558 |
| solid cancers excl. lung | NA | –0.48 (–1.61, 0.65) | 0.37 (–0.69, 1.44) | –0.03 (–0.98, 0.91) | 0.19 (–0.59, 0.97) | 0.889 |
| lung cancer | NA | 0.53 (–1.16, 2.21) | 1.83 (0.22, 3.44) | –0.50 (–2.01, 1.00) | –0.32 (–1.66, 1.01) | 0.264 |
| leukemias excl. CLL | 0.10 (–7.56, 7.76) | 1.90 (–5.58, 9.38) | 3.70 (–4.15, 11.6) | 7.58 (–0.36, 15.5) | –0.20 (–5.26, 4.86) | 0.724 |
| CML | 2.16 (–18.8, 23.1) | 6.79 (–20.3, 33.8) | NC | NC | NC | NC |
| AML | 0.33 (–9.49, 10.2) | –0.92 (–16.4, 14.6) | 0.73 (–7.16, 8.62) | 1.54 (–5.12, 8.21) | 0.26 (–6.65, 7.16) | 0.923 |
| AML+MDS | 0.48 (–9.43, 10.4) | –0.92 (–13.8, 12.0) | 0.17 (–6.74, 7.08) | 1.12 (–4.53, 6.77) | 2.80 (–1.77, 7.38) | 0.887 |
| MM | 0.89 (–12.9, 14.7) | 2.13 (–7.17, 11.4) | 1.77 (–5.33, 8.88) | –0.85 (–7.16, 5.46) | –0.91 (–4.79, 2.98) | 0.381 |
| NHL | NA | –0.92 (–6.08, 4.24) | 0.67 (–4.09, 5.44) | 0.31 (–4.47, 5.09) | 0.62 (–3.28, 4.53) | 0.963 |

^1^ Estimates below the boundary (i.e., ERR < –1) that result from linear extrapolation are reported without censor.

^2^ Test of the homogeneity of windows, where *P* is the p-value for the reported likelihood ratio test statistic and is evaluated under a Chi-square distribution with *k*-1 degrees of freedom for a model with *k* dose parameters.

Abbreviations: CI, confidence interval; CLL, chronic lymphocytic leukemia; CML, chronic myeloid leukemia; ERR, excess relative rate; MDS, myelodysplastic syndrome; MM, multiple myeloma; NA, not applicable; NC, not calculable; NHL, non-Hodgkin lymphoma.

# Table S5. *Post hoc* solid cancer analyses: linear ERR per Gy (90% CI) by time since exposure (TSE) in INWORKS.

| Cohort | Model 1^1^ |  | Model 2 | | |
| --- | --- | --- | --- | --- | --- |
|  | 10–40 |  | 1040 | 40+ | *P* ^2^ |
| full | 0.63 (0.35, 0.93) |  | 0.63 (0.35, 0.93) | –0.02 (–0.57, 0.58) | 0.124 |
| 1958+ | 1.28 (0.75, 1.81) |  | 1.23 (0.72, 1.78) | 0.6 (–0.78, 2.15) | 0.525 |

^1^ Model with single window of exposure (time since exposure between 10–40 years).

^2^ Test of the homogeneity of two windows (TSE 10<40, 40+ years), where *P* is the p-value for the reported likelihood ratio test statistic and is evaluated under a Chi-square distribution with 1 degree of freedom.

Abbreviations: CI, confidence interval; ERR, excess relative rate.

# Table S6. Linear ERR per Gy by age at exposure (AE) in INWORKS restricted to workers hired in 1958 and later.^1^

| Outcome | Lag  (years) | ERR per Gy (90% CI) by age at exposure (years) | | | *P* ^2^ |
| --- | --- | --- | --- | --- | --- |
|  |  | <35 | 3550 | 50+ |  |
| solid cancers | 10 | 0.49 (–0.51, 1.49) | 1.59 (0.75, 2.43) | 1.07 (–0.03, 2.17) | 0.440 |
| solid cancers excl. lung | 10 | 0.69 (–0.48, 1.85) | 1.22 (0.26, 2.18) | 1.48 (0.15, 2.81) | 0.723 |
| lung cancer | 10 | 0.03 (–1.92, 1.98) | 2.55 (0.87, 4.23) | –0.07 (–2.06, 1.92) | 0.219 |
| leukemias excl. CLL | 2 | 1.96 (–4.02, 7.95) | 0.91 (–3.76, 5.57) | 5.63 (–2.93, 14.2) | 0.725 |
| CML | 2 | 7.41 (–14.5, 29.3) | 14.3 (–8.72, 37.3) | 11.8 (–16.9, 40.5) | 0.940 |
| AML | 2 | 2.43 (–4.94, 9.80) | –1.10 (–6.59, 4.39) | 3.97 (–5.52, 13.5) | 0.720 |
| AML+MDS | 2 | 2.33 (–4.63, 9.29) | 2.67 (–3.49, 8.84) | 3.61 (–3.97, 11.2) | 0.976 |
| MM | 2 | 2.40 (–4.00, 8.80) | –0.70 (–5.09, 3.70) | 4.58 (–5.63, 14.8) | 0.705 |
| NHL | 10 | 0.61 (–3.51, 4.74) | –1.10 (–4.30, 2.10) | 1.08 (–3.14, 5.29) | 0.426 |

^1^ Estimates below the boundary (i.e., ERR < –1) that result from linear extrapolation are reported without censor.

^2^ Test of the homogeneity of windows, where *P* is the p-value for the reported likelihood ratio test statistic and is evaluated under a Chi-square distribution with 2 degrees of freedom.

Abbreviations: CI, confidence interval; CLL, chronic lymphocytic leukemia; CML, chronic myeloid leukemia; ERR, excess relative rate; MDS, myelodysplastic syndrome; MM, multiple myeloma; NHL, non-Hodgkin lymphoma.

# Table S7. Linear ERR per Gy by age at exposure (AE) in INWORKS restricted to workers hired prior to 1958.^1^

| Outcome | Lag  (years) | ERR per Gy (90% CI) by age at exposure (years) | | | *P* ^2^ |
| --- | --- | --- | --- | --- | --- |
|  |  | <35 | 3550 | 50+ |  |
| solid cancers | 10 | –0.15 (–0.92, 0.61) | 0.26 (–0.25, 0.78) | 0.28 (–0.45, 1.02) | 0.744 |
| solid cancer excl. lung | 10 | –0.07 (–0.96, 0.82) | 0.12 (–0.50, 0.73) | 0.08 (–0.79, 0.95) | 0.974 |
| lung cancer | 10 | –0.38 (–1.92, 1.16) | 0.61 (–0.38, 1.60) | 0.87 (–0.54, 2.29) | 0.591 |
| leukemias excl. CLL | 2 | 0.22 (–5.56, 6.01) | 4.58 ( 0.48, 8.68) | 2.43 (–2.10, 6.96) | 0.683 |
| CML | 2 | –1.46 (–56.0, 53.0) | 11.4 (–1.01, 23.9) | 7.42 (–6.74, 21.6) | 0.304 |
| AML | 2 | 2.72 (–5.09, 10.5) | 1.36 (–3.57, 6.29) | –1.03 (–5.58, 3.52) | 0.735 |
| AML+MDS | 2 | 3.13 (–3.14, 9.39) | 1.31 (–2.98, 5.59) | –0.85 (–5.37, 3.68) | 0.648 |
| MM | 2 | –0.06 (–6.40, 6.28) | –0.93 (–4.43, 2.58) | 8.41 ( 0.85, 16.0) | 0.194 |
| NHL | 10 | 0.17 (–3.90, 4.25) | 1.61 (–1.32, 4.54) | –2.13 (–4.96, 0.69) | 0.331 |

^1^ Estimates below the boundary (i.e., ERR < –1) that result from linear extrapolation are reported without censor.

^2^ Test of the homogeneity of windows, where *P* is the p-value for the reported likelihood ratio test statistic and is evaluated under a Chi-square distribution with 2 degrees of freedom.

Abbreviations: CI, confidence interval; CLL, chronic lymphocytic leukemia; CML, chronic myeloid leukemia; ERR, excess relative rate; MDS, myelodysplastic syndrome; MM, multiple myeloma; NHL, non-Hodgkin lymphoma.

# Table S8. Post hoc solid cancer analyses: linear ERR per Gy by age at exposure (AE) in INWORKS.

| Cohort | ERR per Gy (90% CI) by AE (years) | | *P* ^1^ |
| --- | --- | --- | --- |
|  | <35 | 35+ |  |
| full | 0.02 (–0.55, 0.63) | 0.64 (0.34, 0.95) | 0.161 |
| 1958+ | 0.53 (–0.42, 1.57) | 1.39 (0.82, 2.02) | 0.248 |

^1^ Test of the homogeneity of windows, where *P* is the p-value for the reported likelihood ratio test statistic and is evaluated under a Chi-square distribution with 1 degree of freedom.

Abbreviations: CI, confidence interval; ERR, excess relative rate.

# Table S9. Linear ERR per Gy by attained age (AA) in INWORKS restricted to workers hired in 1958 and later.^1^

| Outcome | Lag  (years) | ERR per Gy (90% CI) by attained age (years) | | | *P* ^2^ |
| --- | --- | --- | --- | --- | --- |
|  |  | <60 | 6080 | 80+ |  |
| solid cancers | 10 | 1.19 (–0.13, 2.51) | 0.96 (0.39, 1.52) | 2.04 (0.71, 3.36) | 0.424 |
| solid cancers excl. lung | 10 | 1.92 (0.28, 3.57) | 0.78 (0.14, 1.42) | 1.95 (0.52, 3.37) | 0.287 |
| lung cancer | 10 | –0.76 (–2.82, 1.30) | 1.43 (0.29, 2.57) | 2.54 (–0.96, 6.04) | 0.296 |
| leukemias excl. CLL | 2 | –1.10 (–6.98, 4.79) | 2.77 (–1.12, 6.67) | 4.26 (–2.99, 11.5) | 0.445 |
| CML | 2 | 7.66 (–13.9, 29.3) | 8.75 (–4.94, 22.4) | 42.9 (–40.7, 126.5) | 0.543 |
| AML | 2 | –1.10 (–9.19, 7.00) | –0.76 (–4.44, 2.93) | 4.13 (–3.91, 12.2) | 0.307 |
| AML+MDS | 2 | –1.10 (–8.46, 6.27) | 1.07 (–2.94, 5.09) | 9.75 (–0.06, 19.6) | 0.195 |
| MM | 2 | 0.82 (–9.06, 10.7) | –0.10 (–2.81, 2.61) | 13.7 (–4.40, 31.8) | 0.200 |
| NHL | 10 | –0.41 (–5.38, 4.57) | –0.51 (–2.42, 1.41) | 4.14 (–2.38, 10.7) | 0.388 |

^1^ Estimates below the boundary (i.e., ERR < –1) that result from linear extrapolation are reported without censor.

^2^ Test of the homogeneity of windows, where *P* is the p-value for the reported likelihood ratio test statistic and is evaluated under a Chi-square distribution with 2 degrees of freedom.

Abbreviations: CI, confidence interval; ERR, excess relative rate; MDS, myelodysplastic syndrome; MM, multiple myeloma; NHL, non-Hodgkin lymphoma.

# Table S10. Linear ERR per Gy by attained age (AA) in INWORKS restricted to workers hired prior to 1958.^1^

| Outcome | Lag  (years) | ERR per Gy (90% CI) by attained age (years) | | | *P* ^2^ |
| --- | --- | --- | --- | --- | --- |
|  |  | <60 | 6080 | 80+ |  |
| solid cancers | 10 | –0.36 (–1.35, 0.62) | 0.15 (–0.19, 0.49) | 0.37 (–0.17, 0.91) | 0.586 |
| solid cancer exc. Lung | 10 | –0.30 (–1.41, 0.82) | –0.08 (–0.47, 0.32) | 0.38 (–0.22, 0.98) | 0.501 |
| lung cancer | 10 | –0.65 (–2.92, 1.62) | 0.60 (–0.06, 1.26) | 0.33 (–0.91, 1.56) | 0.707 |
| leukemias excl. CLL | 2 | 2.89 (–3.36, 9.14) | 1.62 (–0.87, 4.11) | 6.33 (0.93, 11.7) | 0.347 |
| CML | 2 | 5.58 (–7.14, 18.3) | 5.00 (–3.12, 13.1) | 39.8 (–17.9, 97.5) | 0.222 |
| AML | 2 | 0.79 (–5.34, 6.91) | –0.82 (–3.54, 1.90) | 1.19 (–2.51, 4.88) | 0.487 |
| AML+MDS | 2 | 0.91 (–5.33, 7.16) | –0.82 (–2.80, 1.16) | 2.08 (–0.91, 5.08) | 0.257 |
| MM | 2 | 1.07 (–3.65, 5.79) | 2.17 (–0.64, 4.99) | –0.82 (–3.22, 1.57) | 0.360 |
| NHL | 10 | 8.54 (–2.59, 19.7) | –0.02 (–1.57, 1.53) | –0.50 (–2.39, 1.40) | 0.204 |

^1^ Estimates below the boundary (i.e., ERR < –1) that result from linear extrapolation are reported without censor.

^2^ Test of the homogeneity of windows, where *P* is the p-value for the reported likelihood ratio test statistic and is evaluated under a Chi-square distribution with 2 degrees of freedom.

Abbreviations: CI, confidence interval; ERR, excess relative rate; MDS, myelodysplastic syndrome; MM, multiple myeloma; NHL, non-Hodgkin lymphoma.


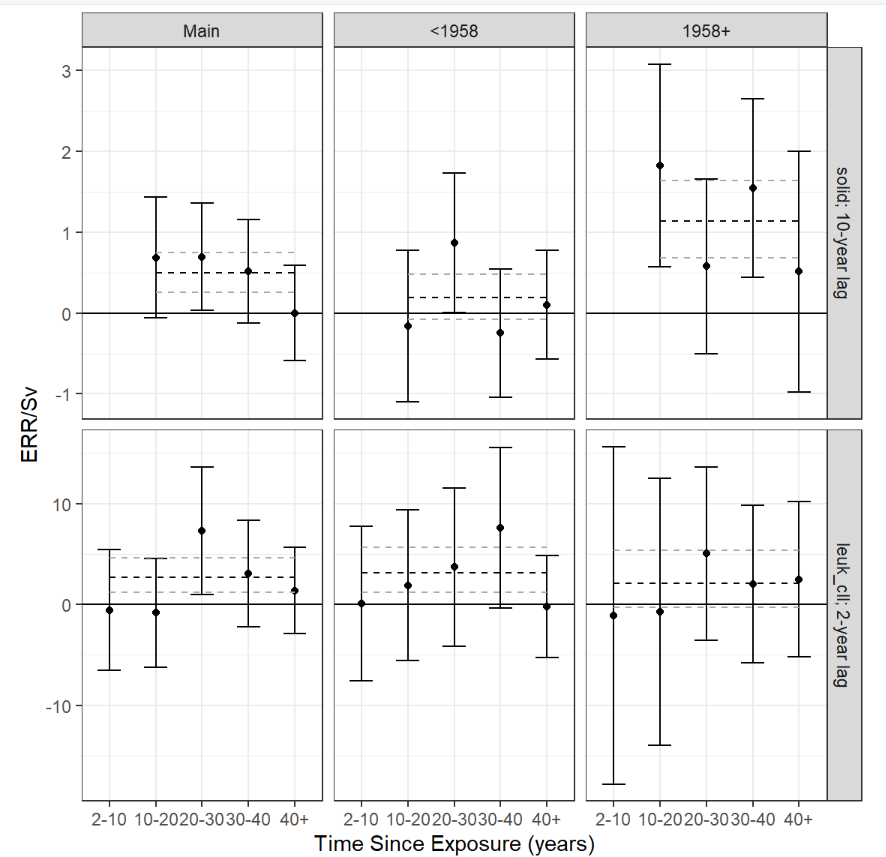


Figure S1. The ERR Gy–1 (filled circles) and associated 90% CIs (whiskers) for categories of time since exposure (TSE) for all solid cancers and leukemia excluding CLL. Results from full (main) and restricted (<1958 and 1958+) cohorts shown in left and right panels, respectively. Bolded dashed line represents the overall estimate. Grey dashed line indicates upper and lower 90% bounds on the overall estimate.


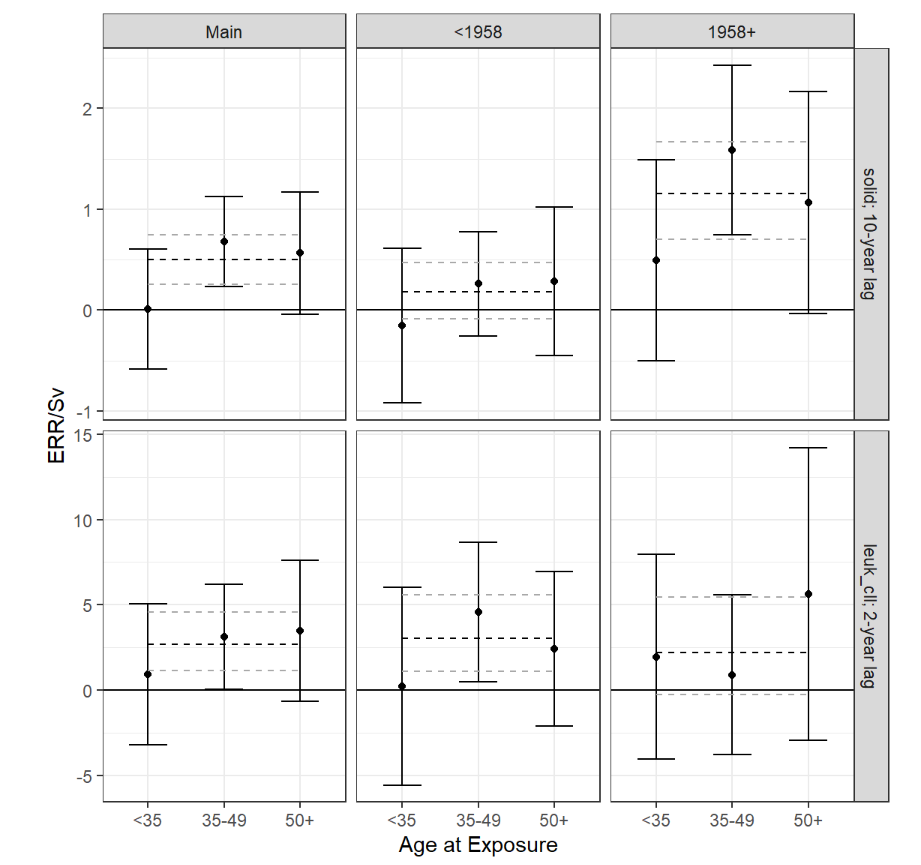


Figure S2. The ERR Gy–1 (filled circles) and associated 90% CIs (whiskers) for categories of age at exposure (AE) for all solid cancers and leukemia excluding CLL. Results from full (main) and restricted (<1958 and 1958+) cohorts shown in left and right panels, respectively. Bolded dashed line represents the overall estimate. Grey dashed line indicates upper and lower 90% bounds on the overall estimate.


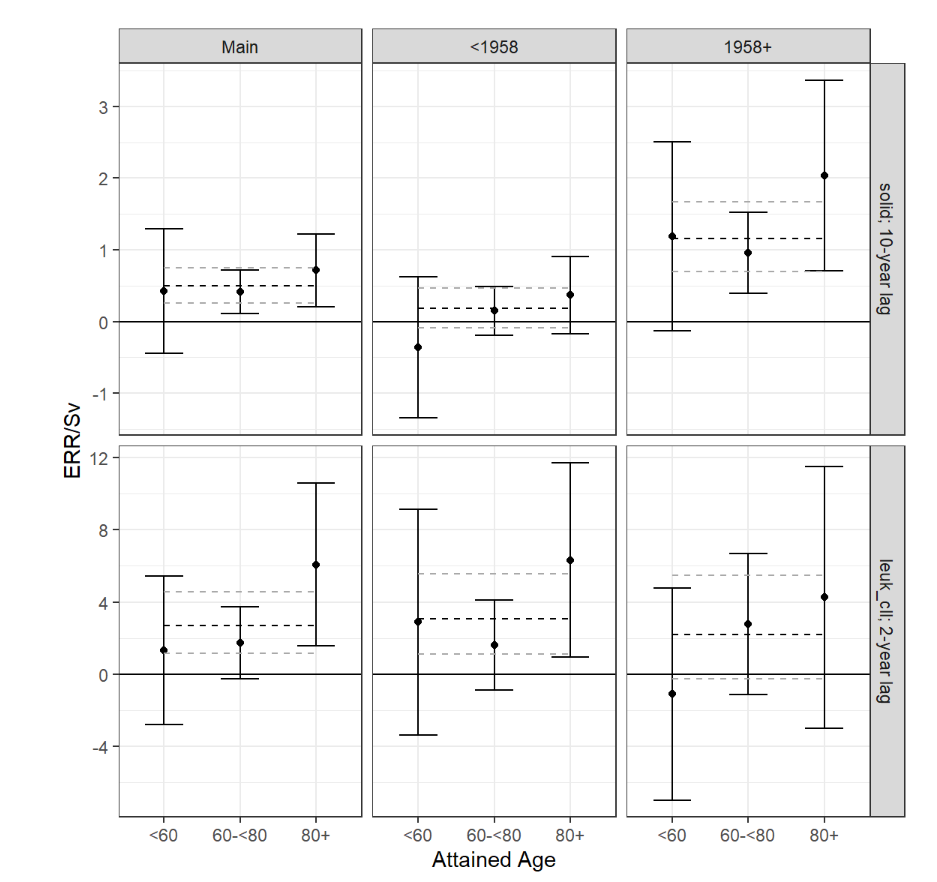


Figure S3. The ERR Gy–1 (filled circles) and associated 90% CIs (whiskers) for categories of attained age for all solid cancers and leukemia excluding CLL. Results from full (main) and restricted (<1958 and 1958+) cohorts shown in left and right panels, respectively. Bolded dashed line represents the overall estimate. Grey dashed line indicates upper and lower 90% bounds on the overall estimate.
